# Mathematical Analysis of a Modified Closed-Form Formula for Design a Uniform Leaky-Wave Antenna With Ultra-Low SLL

**DOI:** 10.1038/s41598-019-44967-w

**Published:** 2019-06-28

**Authors:** A. Kiani, F. Geran, S. M. Hashemi, K. Forooraghi

**Affiliations:** 1 Faculty of Electrical Engineering, Shahid Rajaee Teacher Training University, Tehran, Iran; 2 Department of Electrical and Computer Engineering, Tarbiat Modares University, Tehran, 14155-4838 Iran

**Keywords:** Electrical and electronic engineering, Physics

## Abstract

Straight long slots have high side-lobes in the far-field amplitude patterns, which reduces their use as high-performance antennas. To reduce these side-lobes, a long slot may be tapered to produce the desired radiation patterns. The theory of control of the aperture distribution to reduce side-lobes has been already reported in some works and well known for already some decades. It is, however, shown in this paper that it may not be good enough to achieve ultra-low side lobes. The theory to analyze and design tapered leaky-wave antennas is described in this paper. Since it is very challenging to achieve a mathematical equation in this regard, some parameters will be calculated using simulation in the first step and the shape of the antenna field is obtained based on these parameters. In the next step, a differential equation is derived for the first step parameters. The solution of this differential equation which is the main motivation of this paper will be expressed in three ways where each part is more accurate than the previous one. According to the measurement results, the structure has a side-lobe level more than −45 dB.

## Introduction

The history of creating a slot in a rectangular waveguide as a radiative element dates back to World War II, and the work of Watson, Steveson at McGill University of Canada^1,2^. They found that if a small slot is created in the waveguide wall, electromagnetic waves will radiate into the outer space of the waveguide, therefore, it can be used as an antenna. The first known LWA was the slotted rectangular waveguide, introduced by W. W. Hansen in 1940^3^. The properties of leaky waves were originally derived from the pioneering work of Tamir and Oliner in the late 1950s and early 1960s. This was followed by the extensive development of leaky wave theory and application to these antennas^4,5^. Today, these antennas are used to construct linear and nonlinear arrays. These antennas are suitable for producing a fan beam with low SLL and are used in many strategic telecommunication and radar applications. In many cases, the design of these antennas in the substrate integrated waveguide (SIW) and patch structures are also discussed^6–13^.

LWAs can be divided into three important categories namely, uniform, quasi-uniform and periodic, depending on the type of guiding structure. A uniform structure has a single along the slot in the structure, usually in the form of a waveguide that has been partially opened to allow radiation to occur. The guided wave on the uniform structure is a fast wave and it can only scan the forward quadrant of space, also the quasi-uniform LWA consists of multiple closely spaced slots, this antenna can only scan the forward quadrant. A periodic LWA structure consists of multiple slots that are separated from each other by a greater distance than the quasi-uniform structure and supports a slow wave that is periodically modulated. By selecting a proper distance between slots, it can also scan the forward quadrant in addition to backward quadrant^14,15^.

One of the important features of the LWA is that the distribution of the surface current is easily controlled by the location of the slot. This antenna also offers some other interesting features such as high efficiency, easy feeding design, and high mechanical strength. Thanks to these features, despite the design and construction of a variety of new antennas, LWA is still used in many applications and much research has been conducted on this topic^16–21^. The LWA is an antenna that uses a traveling wave in its structure. To be more exact, this antenna uses a fast wave in its structure in which the phase constant *β* is less than the free-space wavenumber *k*_0_. Therefore, it leaks the power over its structure. Due to the leakage of power, the propagation wavenumber *k*_*x*_ = *α* + *jβ* (*α* is a leakage constant) is complex in the antenna structure. The value of *α* depends on the amount of leakage along the antenna. A large *α* represents a high leakage across the antenna. In this case, the shape of the slot on the antenna does not really have a considerable effect on the radiation field. It means that there is no much control over the amount of SLL. Also, the antenna’s 3 dB beamwidth will be increased. On the other hand, if the amount of *α* is small, the slot length will be effective and the length of the antenna should be selected larger to obtain a low SLL. Also, in this case, the ability to create a narrow beam pattern in the antenna will be provided. Under normal conditions, the antenna is designed to radiate about 90% of the input power and 10% of the remaining power at the end of the antenna is absorbed by a match load. In this case, typically the antenna length is about 10 *λ*, where *λ* is a frequency wavelength. Since the phase constant *β* changes with the frequency, the antenna properties will also change. Some of these variations include the change in 3 dB bandwidth, SLL, antenna gain and angle that is used to scan the space^15,22,23^. In the straight long slots, the power leaks out uniformly, so one drawback of the uniform long straight slot is its high SLL. Control over the beam shape and SLL may be obtained by using an appropriate aperture tapering. Amplitude tapering is one of the most common methods which reduced the SLL by changing the shape, length or width of the slots. Another method is space tapering, in this method by changing the space between the slots of the antenna, SLL is reduced^24,25^. Low SLL is an important factor to minimize the interference of the antenna with other components. Decreasing SLL will make the telecommunication system more efficient in measuring the angle of entrance signals in the antenna pattern, so it is generally desirable to decrease the amount of the SLL.

One of the most important works on the design of the LWA is F. L. Whetten, C. A. Balanis papers were presented in 1991 and 1996 respectively^26,27^. In these works, it is shown that, if the tapering is used, the amount of SLL will greatly improve. But, because of this improvement, what is said is that The exact meander contour is usually very difficult to determine, and therefore is given in this paper only as a sequence of data points^26^.

Another important work has been done by A. A. Oliner based on Hines researches^28,29^. In this paper, the slot on the antenna is expressed by an equation, that is very useful for the design of LWAs. Besides, the authors have also described the drawbacks of this equation. The drawbacks indicate that the equations are not accurate enough and need to be improved further. The main motivation of this paper is to provide more precise equations for the slot shape of the LWA, which can be achieved ultra-low SLL based on these equations. The suggested method, based on both analytical equation and simulation yields optimal SLL compared with previous methods. Drawing the exact field shape on the antenna slot and obtaining the *α* and *β* parameters based on it, is also one of the important achievement of this paper.

## Control of Aperture Distribution

**Figure 1:**
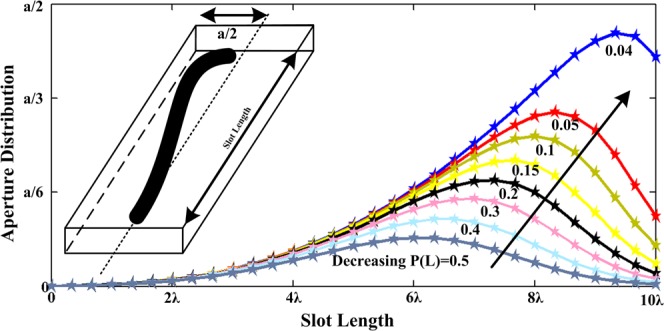
Deformation of the slot on the antenna by reducing the output power.

The design of a leaky wave antenna with length L must be based on *α* and *β*. The angle between the far-field and antenna structure can be determined by using the value of *β* by (theoretical radiation pattern has a maximum at an elevation angle sin(*θ*) = *β*/*K*_0_)^28,29^. The amount of *α* causes the leakage of power to the outside of the antenna. The values of *α* and *β* depend on frequency, antenna geometry, antenna material, the slot width, and length. The *α* and *β* are not constant and will vary over the antenna width and length (which will be shown in the future). To achieve the desired SLL in the antenna pattern base on A. A. Oliner formula, the slot shape is expressed as^28,29^

1 $$2\alpha (y)=\frac{|A{(y)}^{2}|}{\frac{P(0)}{P(0)-P(L)}{\int }_{0}^{L}{|A(\zeta )|}^{2}\,d\zeta -{\int }_{0}^{y}{|A(\zeta )|}^{2}\,d\zeta }$$

where *P*(0) is the input power, at *x* = 0, and *P*(*L*) the output power. *ζ* is the integration variable. Also $\frac{P(0)}{P(0)-P(L)}{\int }_{0}^{L}{|A(\zeta )|}^{2}d\zeta$ is a positive and constant factor. Notice that the unit for *α*(*y*) is neper per unit length. To obtain *α*(*y*) in decibels per unit length, it is multiplied by 8.68. From (1) it can be observed that *α*(*y*) becomes very large for points near to the end of the waveguide wall for *P*(*L*) ≈ 0. This is the main reason that it is common for $\frac{P(L)}{P(0)}$ to be equal to 0.1 and the remaining power being absorbed in a matched load to avoid the presence of any back-lobe. Figure 1 shows a Gaussian slot on the top wall surface of the antenna based on Eq. (1). According to this figure, the following points can be expressed:

1. If we select the antenna width *a*, it is not clear what the maximum of *α*(*y*) is? and how much it can be increased?
2. Knowing the complex propagations constant, which includes phase constant *β* and leakage constant *α*, it is straightforward to design LWA, these parameters do not exist in the equation.
3. The thickness of the slot *w*, is not considered in this case. This factor highly affects the values of *α* and *β*. At a slot width of 0.1λ or less, only the TE surface wave mode can propagate, but at a thickness slot width of 0.2*λ* both the first TM and TE surface wave modes can propagate^27^.
4. The value of *P*(*L*) cannot be zero because the value of *α*(*y*) tends to infinity.

**Figure 2:**
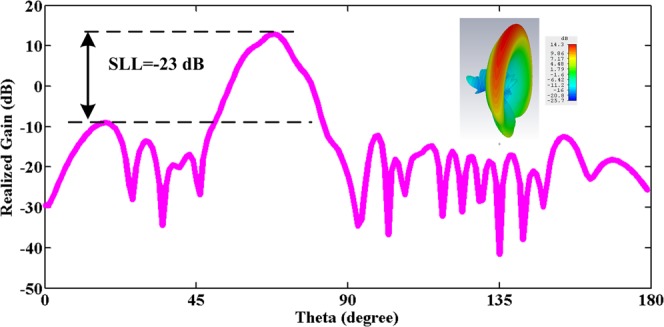
Pattern of a LWA simulated with CST based on formula 1.

Figure 2 illustrates the far-field pattern of the antenna using (1), in which the dimensions of the antenna, operating frequency and materials will be the same as the design process that will be discussed later. All simulations are performed at operating frequency 10 GHz, *w* = 2 mm, *t* = 1 mm, *a* = 17 mm, *b* = 4 mm and slot length *L* = 325 mm. For the use of Eq. 1 and its implementation, various methods have been presented in various papers. In this paper^18,19^ are used to implement Eq. 1. In Fig. 2, the antenna SLL is about −23 dB, while theoretically, the SLL of the far-field pattern must be about −55 dB. The reason for the difference is the above mentioned items.

## Expression of Relationship that are Required for Design LWA

For the proper operation of an antenna, it should be designed as a single mode antenna, usually designed in the *TE*_10_ mode. First, the waveguide dimensions *a*, *b* and length *L* are selected respectively. Then, a very narrow slot (slot width <0.1*λ*) is created on the waveguide. The transverse surface currents density *J*_*y*_ on the upper broad wall is given by (2). *J*_*x*_ and *J*_*z*_ values are very small and can be ignored^30^.

2 $${J}_{y}={A}_{10}\,\sin ({\beta }_{y}y)\exp (-\,j{\beta }_{x}x)$$

where

$${\beta }_{y}=\frac{\pi }{a},\,{\beta }_{x}=\frac{2\pi }{{\lambda }_{g}}=\sqrt{{k}^{2}-{\beta }_{y}^{2}},\,{K}_{0}=\frac{2\pi }{\lambda }=\frac{\omega }{c},$$

$${\lambda }_{g}=\frac{\lambda {\lambda }_{c}}{\sqrt{{\lambda }_{c}^{2}-{\lambda }^{2}}},\,{\lambda }_{c}=2a,\,-\,a/2 < y < a/2$$

**Figure 3:**
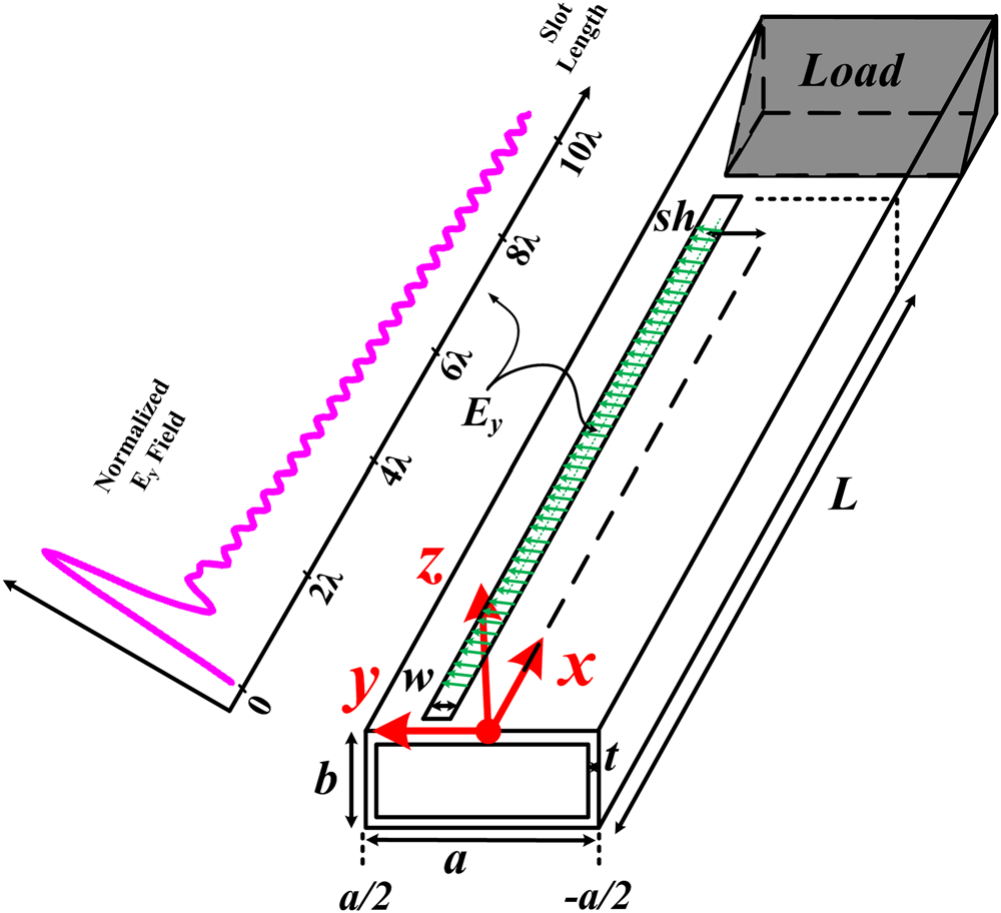
LWA structure used to plot the field shape of the slot.

The shape of a LWA is shown in Fig. 3. Let’s assume the antenna length *L*, the waveguide thickness is *t*, the waveguide dimensions *a*, *b*. and $b < \frac{a}{2}$ to prevent the appearance of other modes. Some of the power will be leaked by the slot and what will remain is absorbed by Emerson and Cuming MF124 absorbent. *w* is the slot width and selected based on the *S*_12_ scattering parameter and the value of *λ*. IF the *w* is large, the radiation power will go up further, and therefore the power absorbed at the end of the waveguide length will be negligible. On the other hand, the antenna 3 dB pattern will be wider, and the SLL cannot be controlled correctly. If *w* is small, the radiation power will be decreased and so the power at the end of the antenna length will be increased, therefore, it is needed to increase the antenna length to achieve the desired SLL. If the antenna length does not change, in this case, the unwanted pattern will be created at the −*θ* in the far-field. Also, *w* will change the values of *α* and *β*. To design the antenna, *w* is selected to have a value of *S*_12_ = −10 dB or less of this value. *sh* represented the slot shifted uniformly from center of the waveguide to the edge of the wall of the waveguide. It is expected *E*_*y*_ in the center of the slot

3 $${E}_{y}={E}_{0}\,\sin (\frac{\pi }{a}y)\exp (-\,\gamma x)$$

where

$$-\,a/2 < y < a/2,\,0 < x < L,\,\gamma =\alpha +j\beta $$

**Figure 4:**
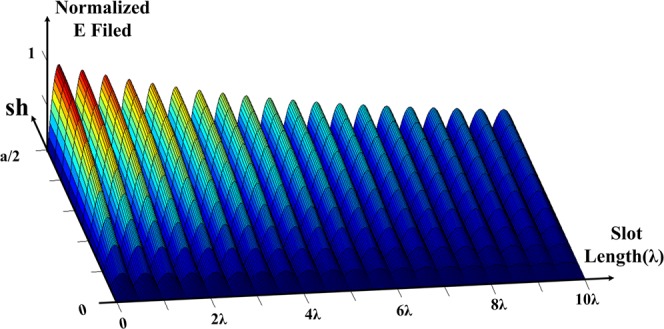
Theoretical shape of the field in the antenna slot.

In the slot of the waveguide, the field will change along the length, in the form of exp(−*γx*), where *γ* = *α* + *jβ* and along the width in the form of, ${E}_{0}\,\sin (\frac{\pi }{a}y)$ for $\frac{-a}{2} < y < \frac{a}{2}$. In this case, *α* is the amount of leakage to the outside and effects of waveguide losses. Figure 4 gives the electric field theoretically for the slot of the waveguide. The radiation field simply depends on $\sin (\frac{\pi }{a}y)$ and exp(−*γx*).

One important point is that these are theoretical relationships and in practice, when the slot is created on the waveguide, these relationships will be changed and the graph of Fig. 4 will not be expected. Since there is no waveguide material, the slot width and length is affected in this case.

## The *α* and *β* Parameters Achievement

**Figure 5:**
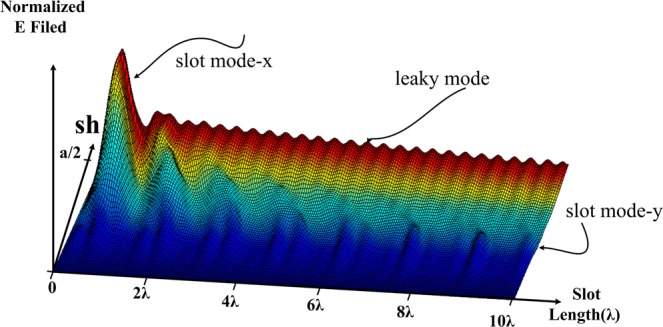
Simulation shape of the field in the antenna slot based on CST.

In the design process, knowing the values of the parameters *α* and *β* are necessary. Therefore, in this section, we will discuss how to obtain the *α* and *β* parameters. The antenna is created in the Computer Simulation Technology - CST software and shift its slot to the end of the wall, and write the *E*_*y*_ field in each section of the parameter *sh* which shown by probes. *E*_*y*_ field can be obtained by a series of probes that lie in the center of the slot. There are *E*_*x*_ and *E*_*z*_ fields but their values are negligible compared to *E*_*y*_ and can be ignored. If the parameter *w* is increased, the values of *E*_*x*_ and *E*_*z*_ will be larger and cannot be neglected. This field of the antenna is shown in Fig. 5. It can be obtained from changing *sh* (from *y* = 0 to *y* = *a*/2) and determining the value of *E*_*y*_ of each probe that stands in the center of the slot.

It is clear in Fig. 5, that the expected sine wave is exponentially changing in the antenna but in two points it is not the case as expected. In the beginning, this anomaly happens when the wave travels into the slot at *sh*&approx;*a*/2, and along the slot at *sh* = *w*/2. In fact, these discontinuities created in the geometric structure have caused this difference. It is clear that this discontinuity will create higher order modes that are called slot mode-x and slot mode-y. Slot mode-y is actually created in order to shift the slot in the direction of the y-axis, and slot mode-x is created to shift the slot in order to enter the field at the beginning of the x-axis. Later it will be shown how to reduce the slot mode-x and its effects on antenna design. In summary

1. The *E*_*y*_ field is zero if the slot is in the center of the antenna.
2. If the slot is at the edge of the antenna wall (*y* = *a*/2), the *E*_*y*_ the field is equal to the maximum value.
3. The field is exponentially decreasing along the antenna, but it shows abnormal behavior in two areas. These two abnormal behaviors can be seen from the comparison between the three-dimensional field shape in a waveguide (Fig. 4) and an antenna with a specific slot on it (Fig. 5).

**Figure 6:**
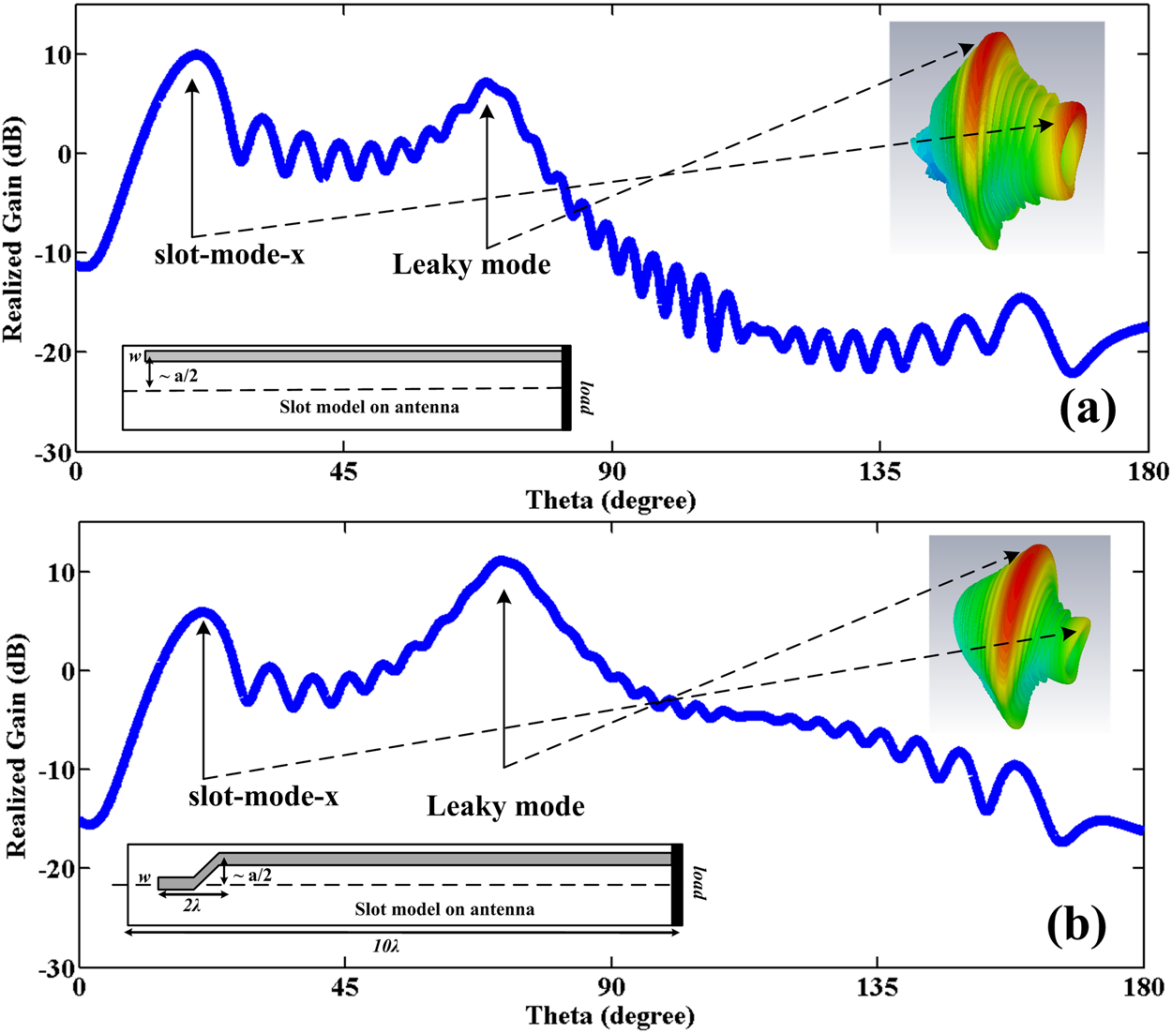
(**a**) Antenna pattern based on leaky and slot modes in straight slot. (**b**) Tapering the shape of the slot around 2*λ* to prevent the slot mode-x, simulated based on CST.

**Figure 7:**
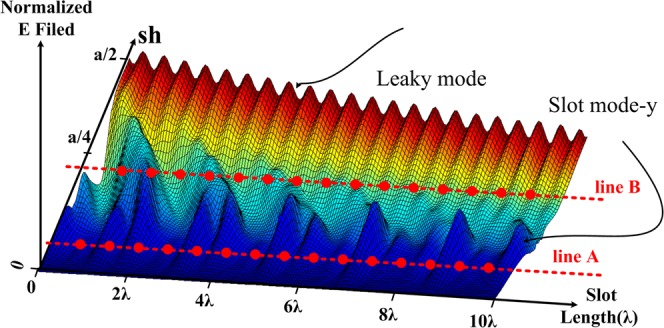
The shape of the field in the LWA slot simulated based on CST after removing the slot mode-x.

As shown in Fig. 5, there are two distinct parts within the shape of the antenna field, slot mode-x and leaky mode. The slot mode-x is created due to the inter of the wave in the slot and disappears at around 2*λ* then the leaky mode starts, which will actually create the original pattern in the antenna. Slot mode-x will increase when the parameter *sh* increases in the antenna, i.e. and the slot moves towards the edge of the antenna wall at the end of the slot shift *sh* = *a*/2 will be greater than the original leaky mode. Also, the slot mode-x will create a pattern near end-fire in the antenna. In fact, since *β* ≈ *k*_0_ at the beginning of the slot, so sin(*θ*) = *β*/*k*_0_ ≈ 1 and a beam near the end-fire will be created in the antenna and there is another beam because of the mode in the original leaky mode. In Fig. 6a,b, these patterns are shown based on these two modes. Note that although the slot mode-x exists in the antenna and causes the antenna pattern, this mode is removed in antenna design. If looking carefully at the three-dimensional field in Fig. 5, it can be found that this mode appears in the values of 0 < *y* = *sh* < *a*/2 while it disappears after around 2*λ*. In the graph of the slot in the following sections, it is shown that for a value of around 2*λ* the value of the slot is approximately *sh* = 0; so this mode must not appear. To verify that the point is correct, Fig. 6b can be used to obtain the exact field graph. In fact, the slot is somehow taper in which the wave limply enters the antenna slot. As can be seen, the effect of the slot mode-x in the far-field pattern has been significantly reduced. The slot mode-x will be created due to the breakdown of the field’s reach into the slot. So, a significant reduction in the value of this mode with a new structure can be expected. By removing the slot mode-x, the shape of the field will be as in Fig. 7. In the case of slot mode-y, this is created when the *sh* parameter is changed, and the value of *sh* ≈ *w*/2 is applied. This means that the full part of the slot passes through the center of the waveguide. This parameter is effective in the design of the antenna and of course, cannot be omitted. Luckily the amplitude of this mode is less than the original leaky mode. If the slot remains in the antenna design around the *sh* ≈ *w*/2, then this parameter will be reduced. The amplitude of this mode is small and can be eliminated by interpolation between line A and line B in Fig. 7.

**Figure 8:**
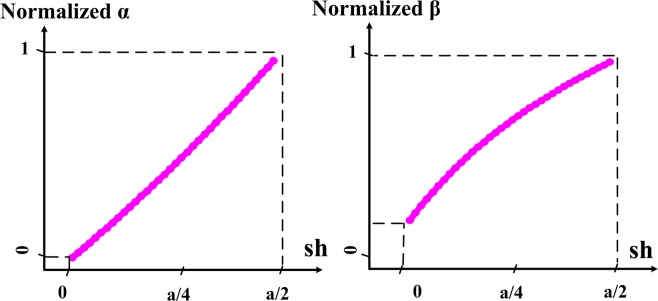
The *α* and *β* parameters in terms of *sh* simulated based on CST.

If only a leaky mode exists, the wave parameters can be calculated. There are a few points in this section. The parameters *α* and *β* in the antenna vary with the change of the parameter *sh*, therefore, the parameters *α* and *β* can be plotted in Fig. 8 in terms of *sh*. It is clear from Fig. 7 that the value of *β* at first (*sh* = 0) is low and will increase at the end of the slot *sh* = *a*/2. That is, the sinusoidal wave is about *sh* = *a*/8 with a period less than the sinusoidal wave at *sh* = *a*/2. The increase of the parameter *α* is also visible from the Fig. 7. The normalized amplitude field does not change much about *sh* = *a*/8, but, in *sh* = *a*/2, the normalized amplitude field decreases more intensely over the antenna length. This means the amount of *α* in the antenna has increased. To perform comparisons with different antennas, these values are normalized. With respect to Fig. 7, it can be concluded that *α* and *β* can be approximated by the (4). Based on Fig. 7 the following points can be considered:

1. As it is known *α*_0_ ≈ 0.
2. Approximate *β*(*y*) with a first-order curve, while a quadratic equation will be a better approximation for it.

4 $$\begin{array}{rcl}\beta (y) & = & {\beta }_{0}+{\beta }_{1}y\\ \alpha (y) & = & {\alpha }_{0}+{\alpha }_{1}y\end{array}$$

Referring to those points, the field in the slot can be written as follows:

5 $${E}_{y}={E}_{0}\,\sin (\frac{\pi }{a}y)\exp (\,-\,\gamma x),\,\gamma =\alpha (y)+j\beta (y)$$

This equation is the basis of the next section. The difference between Eqs (3) and (5) is that, *α* and *β* are not constant and are functions of *y*(*x*) in (5).

## Antenna Design Process

**Figure 9:**
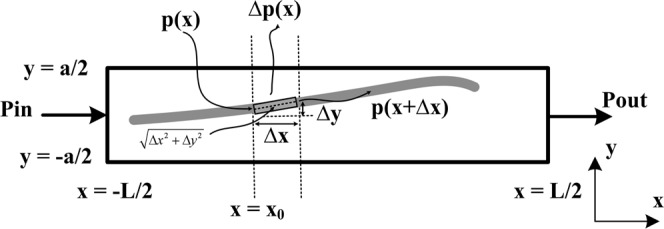
Creating a small differential on the antenna.

This section introduces the mathematical calculations for the LWA to reduce SLL. If created a slot element on the antenna as shown in Fig. 9, a part of the power *p*(*x*) introduced into the element Δ*x* is radiated by the slot Δ*p*(*x*), and the rest travels inside the waveguide *p*(*x* + Δ*x*). Considering ${E}_{y}={E}_{0}\,\sin (\frac{\pi }{a}y)\exp (\,-\,\gamma x)$ where the slot length is in the range of −*L*/2 ≤ *x* ≤ *L*/2, The goal is to find the *f*(*x*) with the minimum possible condition for the SLL. If the slot is narrow, then the following assumptions can be made:

1. The power radiated from the Δ*x* element is proportional to *E*(*x*)^2^ that will be normalized in this equation.
2. The power radiated from the Δ*x* element is proportional to the input power *p*(*x*).
3. The power radiated from the Δ*x* element is proportional to the element size $\sqrt{{\rm{\Delta }}{x}^{2}+{\rm{\Delta }}{y}^{2}}$.

According to the above three hints, the following equation can be written:

6 $$p(x+{\rm{\Delta }}x)-p(x)=Kp(x){\sin }^{2}(\frac{\pi }{a}y)\exp (\,-\,2\gamma x)\sqrt{{\rm{\Delta }}{x}^{2}+{\rm{\Delta }}{y}^{2}}$$

In this equation, *K* is a negative constant. When |*K*| is a large value, this indicates it is more capable of radiation power in the element and passes less power through the Δ*x* element. Note that the units of both sides of the (6) are of power, because ${\sin }^{2}(\frac{\pi }{a}y)\exp (\,-\,2\gamma x)$ is unitless. $\sqrt{{\rm{\Delta }}{x}^{2}+{\rm{\Delta }}{y}^{2}}$ is in *meter*. The unit of *K* is selected in the form of 1/*meter* until the both sides of the Eq. (6) is of power. Divide the both sides of (6) by Δ*x* and apply $\mathop{\mathrm{lim}}\limits_{{\rm{\Delta }}x\to 0}$ to the both sides

7 $$\frac{p(x+{\rm{\Delta }}x)-p(x)}{{\rm{\Delta }}x}=Kp(x){\sin }^{2}(\frac{\pi }{a}y)\exp (-2\gamma x)\sqrt{1+{y^{\prime} }^{2}}$$

Knowing the following mathematical facts

8 $$\{\begin{array}{lllll}\mathop{\mathrm{lim}}\limits_{{\rm{\Delta }}x\to 0}\frac{p(x+{\rm{\Delta }}x)-p(x)}{{\rm{\Delta }}x} & = & \frac{{\rm{\Delta }}p(x)}{{\rm{\Delta }}x} & = & p\text{'}(x)\\ \frac{d}{dx}{\int }_{a}^{x}\,p(x)dx & = & p(x),\,\mathop{\mathrm{lim}}\limits_{{\rm{\Delta }}x\to 0}\frac{{\rm{\Delta }}y(x)}{{\rm{\Delta }}x} & = & y\text{'}\end{array}$$

Based on (7) and (8), it can be concluded that

9 $$\frac{p\text{'}(x)}{p(x)}=K\,{\sin }^{2}(\frac{\pi }{a}y)\exp (\,-\,2\gamma x)\sqrt{1+y{\text{'}}^{2}}$$

The answer to the Eq. (9) based on the appendix A will be as follows

10 $$y(x)=\frac{a}{\pi }{\sin }^{-1}\sqrt{\frac{A{(x)}^{2}\exp (2\gamma x)}{K\int A{(x)}^{2}dx+C}}$$

The unknown parameters of this equation are *K* and *C*. *K* is a negative and *C* is a positive coefficient. By comparing (1) and (10), it can be found that there are many similarities. For example [*A*(*x*)^2^] in the nominator and $\int A{(x)}^{2}dx$ in the denominator in both equations. Since the maximum value of the expression ${\sin }^{-1}\sqrt{\frac{A{(x)}^{2}\exp (2\gamma x)}{K\int A{(x)}^{2}dx+C}}$ is equal to *π*/2, therefore the maximum value of *y*(*x*) is equal to *a*/2. To calculate *K* and *C* let’s consider appendix B.

In a special case for the Gaussian function, one can design a slot on the antenna. The Gaussian distribution is also commonly called the normal distribution and is often described as a bell-shaped curve. *σ* is the standard deviation and *σ*^2^ is the variance.

11 $$A(x)=\frac{1}{\sqrt{2\pi }\sigma }\exp (\frac{-{x}^{2}}{2{\sigma }^{2}}),\,-\,l/2\le x\le l/2$$

By referring to (12)

12 $$erf(\frac{x}{\sigma })=\frac{1}{\sqrt{\pi }\sigma }{\int }_{-x}^{x}\exp (\frac{-{t}^{2}}{{\sigma }^{2}})dt$$

Also based on appendix A, Eq. (30) is a complex and nonlinear equation and should be solved numerically. But it should be noted how much error is acceptable in its approximation matters, thus the following three conditions are considered for solving this equation with the Gaussian function.

### First condition

First, assume that *y*′^2^ = 0 and *γ* = 0. In this case, (30) changes into the following equation.

13 $${\sin }^{2}(\frac{\pi }{a}y).[K\sqrt{\pi }\sigma \,\exp (\frac{{x}^{2}}{{\sigma }^{2}})erf(\frac{x}{\sigma })+2\pi C{\sigma }^{2}\exp (\frac{{x}^{2}}{{\sigma }^{2}})]=1$$

where $$y(x)$$ is

14 $$y(x)=\frac{a}{\pi }{\sin }^{-1}[\sqrt{\frac{\exp (\frac{-{x}^{2}}{{\sigma }^{2}})}{K\sqrt{\pi }\sigma erf(\frac{x}{\sigma })+2\pi C{\sigma }^{2}}}]$$

**Figure 10:**
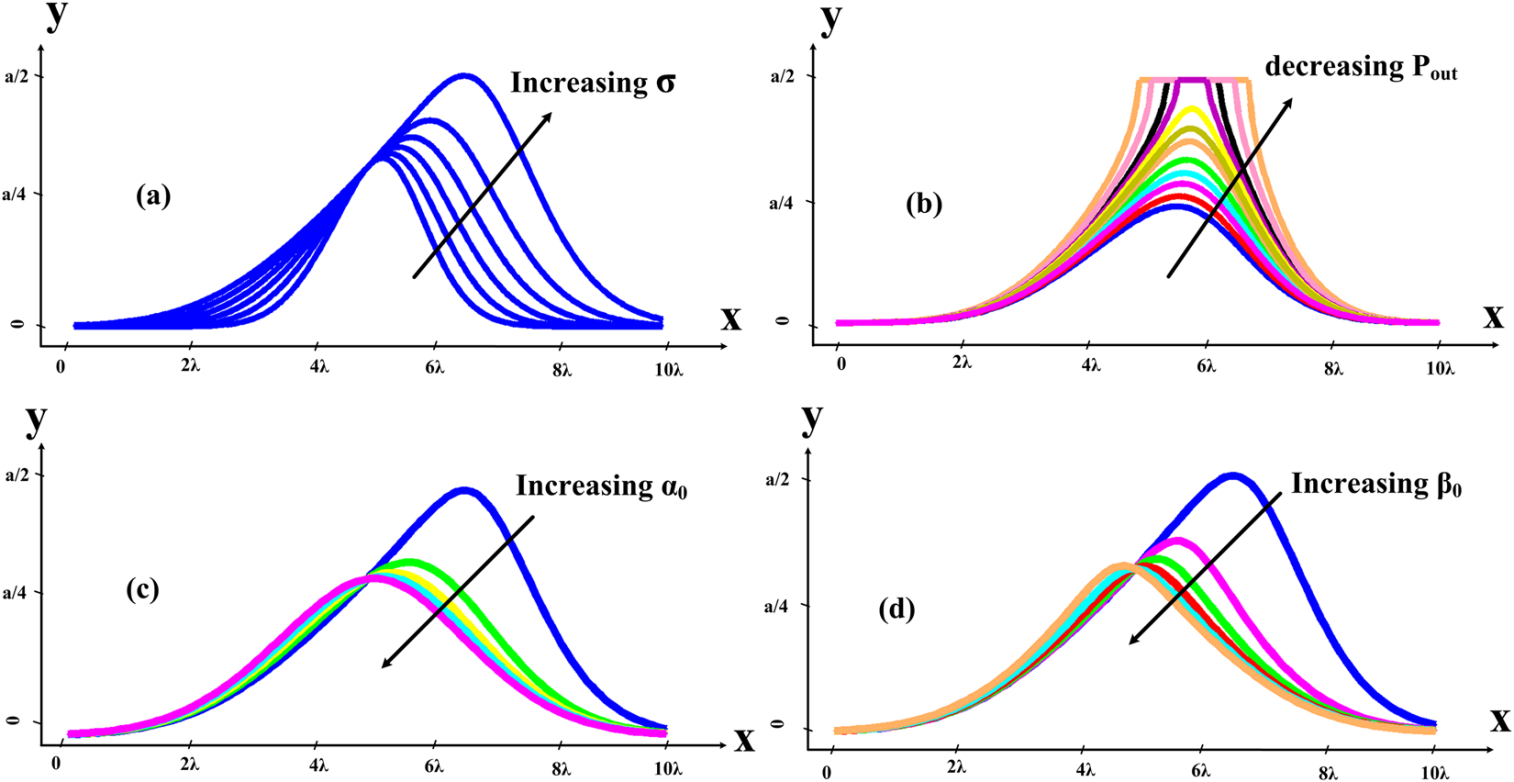
(**a**) Effect of the parameter *σ* in the slot shape. (**b**) Effect of decreasing the output power in the slot shape. (**c**) Effect of increasing the *α*_0_ in the slot shape. (**d**) Effect of increasing the *β*_0_ in the slot shape.

It is important to know that, if *P*_*out*_ = 0, the *K* goes to infinity (see Appendix B, Eq. (34)) and in this case, zero will be one of the obvious answers of the differential equation. In Fig. 10a–d, the curve of (14) is plotted for the given values *K*, *C,* and *σ*. The effect of the parameter *σ* in the slot shape is that the curve slope will increase to higher values by increasing the parameter *σ*. By increasing the parameter *σ*, in reality, the less power of the antenna will leak out and the curve of the slot should be higher. The effect of decreasing the output power on the antenna curve shape is shown in Fig. 10b. The curve is plotted for the given values *K*, *C* and *σ* and decreasing the output power. If the power delivered to the end of the antenna is low, the antenna slot will move toward the antenna edge of the wall. If the shape of the antenna slot at the end of the wall is cut in the value of *a*/2, it shows that with this length, more power will be delivered to the end of the antenna than the selected output power in the Eq. (34). Therefore, in order to eliminate this cut, either the length of the antenna should be increased or the amount of power at the end of the antenna should be decreased.

### Second condition

As the second condition, assume that *γ* = *α*_0_ + *jβ*_0_ where *α* and *β* are constant in the antenna, and also *y*'^2^ = 0, despite, that in the previous sections it was shown that these parameters are not constant and are changed by the width of the slot. In this case, (30) will change into

15 $$y(x)=\frac{a}{\pi }{\sin }^{-1}[\sqrt{\frac{\exp (\frac{-{x}^{2}}{{\sigma }^{2}})\exp (2({\alpha }_{0}+j{\beta }_{0})x)}{K\sqrt{\pi }\sigma erf(\frac{x}{\sigma })+2\pi C{\sigma }^{2}}}]$$

To examine the parameters *α*_0_ and *β*_0_, set each one to zero and examine other effects. First, if sweep *α*_0_ and set *β*_0_ = 0. In this case, the antenna slot will move towards smaller values and will be farther away from the end of the wall (*y* = *a*/2). This means that the power leakage to the outside of the antenna has increased so the length of the antenna can be reduced in this case. If change *β*_0_ and set *α*_0_ = 0 the same behavior from the curve will be observed again. The effects of increasing *β*_0_ and *α*_0_ on the antenna slot indicate that, as these parameters increase, the slot shape changes and moves toward *y*(*x*) with lower values (Fig. 10c,d). Justifying this, with the increase of *α*_0_, the power leakage to the outside the antenna will actually increase and leak out more so there is no need to change the antenna slot to the end of the antenna wall (*y* = *a*/2). When the *α*_0_ is larger, the antenna slot will have a lower range value than *a*/2. The increase of *α*_0_ will be due to an increase in the slot diameter or any factor causing more leakage to the outside of the antenna. An increase of *β*_0_ also yields such an outcome. In fact, the increase of *β*_0_ is equivalent to increasing the antenna length or increasing the frequency, which in any case yields lower output power at the end of the antenna. Thus the slot will move to a smaller amount than *a*/2.

### Third condition

As the third statement, assume that *γ* = (*α*_0_ + *jβ*_0_) + (*α*_1_ + *jβ*_1_)*y*. In this case, (30) must be completely solved. This equation is a nonlinear equation which should be solved using numerical methods. In Table 1 an example of this equation is solved numerically.

**Table 1:** Data points of the slot (*mm*).

*x*	*y*	*x*	*y*	*x*	*y*	*x*	*y*
0	0.000	85	2.0364	170	6.4458	255	6.6260
5	0.0007	90	2.2910	175	6.6399	260	6.3595
10	0.0115	95	2.5532	180	6.8180	265	6.0535
15	0.0253	100	2.8215	185	6.9787	270	5.7066
20	0.0394	105	3.0945	190	7.1206	275	5.3173
25	0.0704	110	3.3709	195	7.2424	280	4.8844
30	0.1270	115	3.6493	200	7.3425	285	4.4063
35	0.2076	120	3.9282	205	7.4198	290	3.8818
40	0.3110	125	4.2064	210	7.4727	295	3.3095
45	0.4358	130	4.4824	215	7.4999	300	2.6879
50	0.5805	135	4.7548	220	7.5000	305	2.0156
55	0.7438	140	5.0223	225	7.4716	310	1.2914
60	0.9243	145	5.2834	230	7.4134	315	0.3186
65	1.1206	150	5.5369	235	7.3239	320	0.0000
70	1.3314	155	5.7812	240	7.2019	325	0
75	1.5552	160	6.0151	245	7.0457	—	—
80	1.7906	165	6.2371	250	6.8542	—	—

## Simulations and Results

In this section, the simulation of the antenna is described. The antenna is designed for operating frequency 10 GHz, *w* = 2 mm, *t* = 1 mm, *a* = 17 mm, *b* = 4 mm and slot length *L* = 325 mm. To see the effects of the three design sections (5.1–5.3), cut the top of the antenna apart and screw it down to the bottom. The following points are very important for building an antenna.

1. The upper part of the antenna is selected from the stainless steel and lower part of it from the aluminum. The reason for this is that the steel exhibits higher resistance to machining than aluminum. In the construction phase, the bottom part of the antenna is made up and screwed up to the upper part so that no displacement occurs, and then the slot is created on the upper part of the antenna (Fig. 11). The reason for this is to change the upper part and build different methods and designs on it. In fact, to change the design method, it’s just enough to re-create the upper part of the antenna.

**Figure 11:**
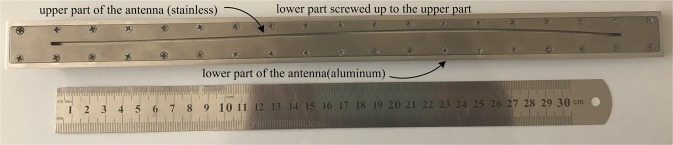
The photograph of the fabricated antenna.
2. Since the high precision is required to create the slot on the antenna, the wire cut machine model DK7740b is used with micron precision.

**Figure 12:**
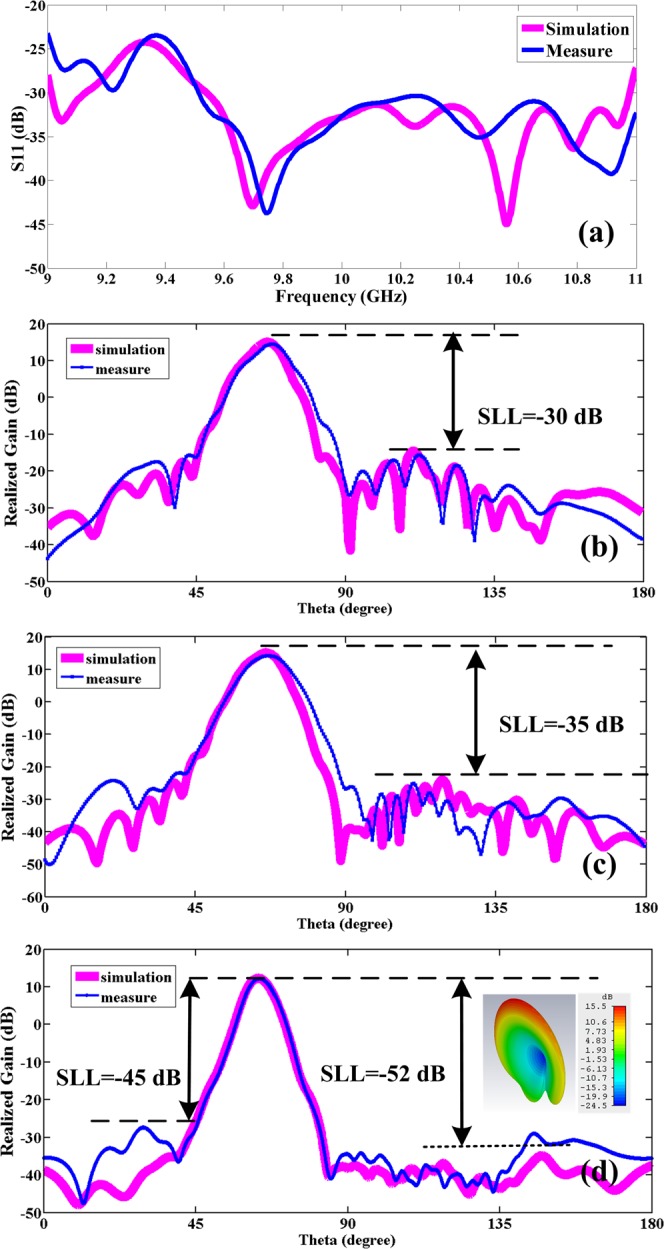
Theoretical and measured *S*_11_ and patterns for the LWAs. (**a**) *S*_11_. (**b**) is based on first condition and Eq. (14). (**c**) is based on second condition and Eq. (15). (**d**) is based on third condition and Table 1.

The practical measurement results are shown in Fig. 12a–d. Since the antenna SLL is very low, it is used to measure it in the transmitter with a Power Amplifier (PA) (before the standard Horn antenna) and in the receiver (after the LWA) with a Low Noise Amplifier (LNA). It is clear from Fig. 12b–d the value of the SLL is about 30 dB and 35 dB in the first and second method based on (14) and (15) respectively.

**Figure 13:**
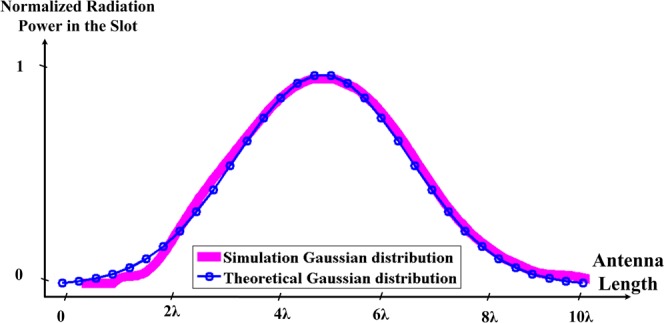
Comparison of the Gaussian distribution generated on the LWA in theory and simulation.

Also, the value of the SLL is about −52 dB in simulation and −45 dB in measurement base on numerical method and data points in Table 1 (Fig. 12d). In fact, we implemented a Gaussian distribution on the antenna. After creating this slot on the antenna, we obtain the radiation power from within the slot and compare it with the Gaussian distribution. This comparison is shown in Fig. 13. The simulation distribution is very close to the theory. The slight difference in the comparison of these two curves at the beginning of the graph is due to the existence of slot mode-x.

## Conclusion

The proposed method for designing a LWA was based on two parts, simulation and mathematical relation. By using this method, at first the aperture field in the slot is calculated, so the antenna material and different conditions of construction and the diameter of the slot are considered. Since changing these parameters will change the *γ* = (*α*_0_ + *jβ*_0_) + (*α*_1_ + *jβ*_1_)*y*(*x*) in the antenna. Here, according to the method described the amount of SLL is significantly improved in comparison to Fig. 2. The design will be performed for a single frequency, but the results are appropriate in the bandwidth of about 1 GHz. It is important to say that the cross- polarization level is about approximately 34 dB down for the designed antenna. A comparison between the three methods described in the paper is shown that the radiation efficiency enhancement by improving the SLL. Also, the results are performed for a Gaussian window, but if other windows are used, other properties can be extracted from the antenna. These properties include antenna gain, 3 dB bandwidth, antenna SLL and isolation between two antennas. Many of the problems with these antennas are that when a radom is placed on the antenna as a coating, the parameters of these antennas will change. This change is so much that the SLL of the antenna will destroy. The reason is that these materials will be placed directly on the slot and will greatly affect the surface current at this point. To improve this problem, it is possible to design the antenna and obtain the parameters *α* and *β* using the antenna coating. Another problem with these antennas is that their pattern will change much when they are on a massive ground. In this case, the antenna can be calculated from the beginning on a massive ground plane and design parameters *α* and *β* using a massive ground considered on the antenna.

## Supplementary information

Supplemental information
