# Publisher Correction: Mathematical Analysis of a Modified Closed-Form Formula for Design a Uniform Leaky-Wave Antenna With Ultra-Low SLL

**DOI:** 10.1038/s41598-020-80762-8

**Published:** 2021-01-04

**Authors:** A. Kiani, F. Geran, S. M. Hashemi, K. Forooraghi

**Affiliations:** 1 Faculty of Electrical Engineering, Shahid Rajaee Teacher Training University, Tehran, Iran; 2 Department of Electrical and Computer Engineering, Tarbiat Modares University, Tehran, 14155-4838 Iran

Correction to: Scientific Reports 10.1038/s41598-019-44967-w, published 28 June 2019.

In the original version of this Article, A. Kiani was incorrectly listed as the corresponding author. The correct corresponding author for this Article is F. Geran. Correspondence and request for materials should be addressed to f.geran@sru.ac.ir.

This error has now been corrected in the HTML and PDF versions of the Article.

