# Supplementary material for: Mathematical Analysis of a Modified Closed-Form Formula for Design a Uniform Leaky-Wave Antenna With Ultra-Low SLL
Source: Sci Rep. 2019 Jun 28;9:9372. doi: 10.1038/s41598-019-44967-w (PMC6599019; doi:10.1038/s41598-019-44967-w)
Supplement: Supplementary file 1 — Supplemental information [file 41598_2019_44967_MOESM1_ESM.pdf]

Mathematical Analysis of a Modified Closed-Form Formula for Design a Uniform Leaky-Wave Antenna With Ultra-Low SLL

A. Kiani*, F. Geran, S. M. Hashemi, K. Forooraghi

March 26, 2019

Appendix A

Integrating both sides of Eq. (9) yields

$$p(x) = P\exp[\int K\sin^2(\frac{\pi}{a}y)\exp(-2\gamma x)\sqrt{1+y'^2}dx] \tag{1}$$

where P is a constant. As a comparison between Eq. (16) and [22-23], the power distribution along the antenna can be expressed in the form of $p(x) = p(0)\exp[-2\int_0^x \alpha(\xi)d\xi]$ in [19,20], where $\alpha(x)$ is corresponding to the aperture amplitude. Substituting (16) into (7) results in

$$\frac{\Delta p(x)}{\Delta x} = KP\underbrace{\sin^2(\frac{\pi}{a}y)\exp(-2\gamma x)\sqrt{1+y'^2}}_{g(x)}. \underbrace{\exp[\int K\sin^2(\frac{\pi}{a}y)\exp(-2\gamma x)\sqrt{1+y'^2}dx]}_{s(x)} \tag{2}$$

To have a low SLL, the amount of radiation power must follow the amplitude distribution in term of coefficients such as ChebyChef, Gaussian, Taylor and so on. Suppose that the desired aperture distribution (which would achieve the specified radiation pattern) is $A(x)$. It can be written:

$$KPg(x)s(x) = -BA(x)^2 \rightarrow \frac{2g(x)s(x)}{A(x)} = -\frac{2B}{KP}A(x) \tag{3}$$

where B is a positive constant for proportionality. Differentiating from (18) gives:

$$g'(x)s(x) + s'(x)g(x) = -\frac{2B}{KP}A(x)A'(x) = \frac{2g(x)s(x)}{A(x)}A'(x) \tag{4}$$

Using Eqs. (9), (16) and (17) gives

$$s(x) = \exp[\int K\sin^2(\frac{\pi}{a}y)\exp(-2\gamma x)\sqrt{1+y'^2}dx] = \frac{p(x)}{P} \tag{5}$$

$$g(x) = \sin^2(\frac{\pi}{a}y)\exp(-2\gamma x)\sqrt{1+y'^2} = \frac{1}{K}\frac{p'(x)}{p(x)} \tag{6}$$

*A. Kiani (e-mail: a.kiani@sru.ac.ir), F. Geran (e-mail: f.geran@sru.ac.ir) and S. M. Hashemi (e-mail: sm.hashemi@sru.ac.ir), Faculty of Electrical Engineering, Shahid Rajaee Teacher Training University, Tehran, Iran

$$s(x) = \exp(\int Kg(x)dx) \tag{7}$$

Differentiating from (22) gives

$$s'(x) = Kg(x)\exp(\int Kg(x)dx) = Kg(x)s(x) \tag{8}$$

From Eqs. (19) and (23), one gets:

$$g'(x)s(x) + Ks(x)g(x)^2 = \frac{2g(x)s(x)}{A(x)}A'(x) \tag{9}$$

By removing $s(x)$ from the sides of (24)

$$\frac{-g'(x)}{g(x)^2} + \frac{2A'(x)}{A(x)}\frac{1}{g(x)} = K \tag{10}$$

Differential equations in form of (26) are called Bernoulli Differential Equations. This is a non-linear differential equation that can be transformed into linear Ordinary Differential Equation (O.D.E) (in this case $n = 2$).

$$g'(x) + P(x)g(x) - Q(x)g(x)^n = 0 \tag{11}$$

By selecting the variable $w(x) = g(x)^{1-n}$ it is solvable. Selecting $w(x) = \frac{1}{g(x)}$, $w'(x) = -\frac{g'(x)}{g(x)^2}$ yields:

$$w'(x) + \frac{2A'(x)}{A(x)}w(x) = K \tag{12}$$

Multiplying the both sides of (27) by $A(x)^2$, we will have:

$$w'(x)A(x)^2 + 2A'(x)A(x)w(x) = KA(x)^2 \tag{13}$$

Therefore

$$\begin{aligned} &[w(x)A(x)^2]' = KA(x)^2 \rightarrow \\ &w(x) = \tfrac{K}{A(x)^2}\int A(x)^2 dx + \tfrac{C}{A(x)^2} \end{aligned} \tag{14}$$

In (29), C is a constant coefficient obtained from the initial conditions of the deferential equation. Use the relation $w(x) = \frac{1}{g(x)}$:

$$\begin{aligned} g(x) &= \sin^2(\tfrac{\pi}{a}y)\exp(-2\gamma x)\sqrt{1+y'^2} \\ &= \frac{1}{[\frac{K}{A(x)^2}\int A(x)^2 dx + \frac{C}{A(x)^2}]} \end{aligned} \tag{15}$$

This differential equation is a nonlinear equation and should be solved using numerical methods. Note that the $\gamma = (\alpha_0 + j\beta_0) + (\alpha_1 + j\beta_1)y(x)$ where γ is a function of $y(x)$. To solve this equation, since the length of the slot is large enough relative to its width, by approximating $y' = 0$, the equation will be as (10).

Appendix B

By integrating both sides of Eq. (9)

$$\int_{x=-L/2}^{x=L/2} \frac{p'(x)}{p(x)}dx = \int_{x=-L/2}^{x=L/2} K\sin^2(\frac{\pi}{a}y)\exp(-2\gamma x)\sqrt{1+y'^2}dx \tag{16}$$

$$\ln p(L/2) - \ln p(-L/2) = K \int_{x=-L/2}^{x=L/2} \sin^2(\tfrac{\pi}{a}y)\exp(-2\gamma x)\sqrt{1+y'^2}dx \tag{17}$$

Use the following relationships to simplify:

$$p(-L/2) = P_{in}, \quad p(L/2) = P_{out}, \quad y'^2 = 0 \tag{18}$$

P_{in} is the input power to the antenna and P_{out} is the remaining power at the antenna end, which is absorbed by the match load.

$$K = \frac{\ln(\frac{P_{out}}{P_{in}})}{\int_{x=-L/2}^{x=L/2} \sin^2(\frac{\pi}{a}y)\exp(-2\gamma x)dx} \tag{19}$$

Because of existence of expression $\ln(\frac{P_{out}}{P_{in}})$, the value of K is negative and due to $\int_{-L/2}^{L/2}[...]dx$ in the denominator of (34) the unit of $|K|$ is $1/m$. The coefficient C is obtained by putting the value $x = L/2$ in (30). Also, approximations of (35) can be used for simplicity.

$$\begin{cases} y'^2 = 0, \int_{x=-L/2}^{x=-L/2} KA(x)^2 dx = 0 \\ y(-L/2) \approx 0 \to \sin^2(\frac{\pi}{a}y(\frac{-L}{2})) \approx (\frac{\pi}{a}y(\frac{-L}{2}))^2 \\ g(\frac{-L}{2}) = (\frac{\pi}{a}y(\frac{-L}{2}))^2 \exp(-2\gamma(\frac{-L}{2})) \end{cases} \tag{20}$$

So

$$(\frac{\pi}{a}y(\frac{-L}{2}))^2 \exp(\gamma L) = \frac{A(\frac{-L}{2})^2}{C} \tag{21}$$

Using the approximation of $A(-L/2)^2 \approx y(-L/2)^2$,

$$C = \frac{(a/\pi)^2}{\exp(\gamma L)} \tag{22}$$

Author Contributions

A. Kiani and F. Geran conceived the idea, analyzed the theoretical feasibility, and wrote the manuscript. S. M. Hashemi carried out the full-wave simulations, designed the samples and performed the measurements.K. Forooraghi contributed to the discussions on theoretical feasibility and design improvements.

Additional Information

Competing interests: The authors declare that they have no competing interests.
